# The Role of the Major Histocompatibility Complex Region on Chromosome 6 in Skin Atrophy: A Mendelian Randomization Study

**DOI:** 10.1111/jocd.70040

**Published:** 2025-03-18

**Authors:** Chenyu Zhao, Zhonghao Fan, Ruihan Zhang, Yuehang Sun, Wen‐Yang Li

**Affiliations:** ^1^ Department of China Medical University‐The Queen's University of Belfast Joint College, School of Pharmacy China Medical University Shenyang China; ^2^ School of Pharmacy Queen's University Belfast Belfast UK; ^3^ Respiratory and Critical Care Department The First Hospital of China Medical University Shenyang China

**Keywords:** aging, chromosome 6, GWAS, skin atrophy

## Abstract

**Background:**

Skin atrophy (SA) is a pathological condition marked by the thinning of the skin, decreased elasticity, and reduced functionality, often arising from aging, chronic glucocorticoid use, or autoimmune diseases.

**Objective:**

This study investigates the role of the major histocompatibility complex (MHC) region on chromosome 6 in the development of SA.

**Methods:**

We applied summary‐data‐based Mendelian randomization (SMR) using eQTL data of three skin‐related tissues (whole blood, lower leg, and suprapubic) from the GTEx database, and SA genome‐wide association study data from FinnGen. Further, we conducted functional enrichment, colocalization, and drug enrichment analyses on the core genes (intersection genes) to explore their functions and druggability.

**Results:**

Six core genes (*PSORS1C3*, *HLA‐C*, *HLA‐DRB5*, *HLA‐DRB6*, *HLA‐DQA1*, and *HLA‐DQB1*) located on chromosome 6p21 were consistently identified across all tissues. Functional enrichment, pathway, and protein–protein interaction analyses revealed that these genes are involved in antigen processing and immune response regulation. Drug enrichment analysis highlighted potential therapeutic targets, including interactions with palladium, azathioprine, and insulin. However, limitations in available data for *PSORS1C3* and *HLA‐DRB6*, as well as inconclusive colocalization results, suggest a need for further research.

**Conclusion:**

This study highlights the involvement of six core genes within the MHC region on chromosome 6 in the development of SA, emphasizing their roles in immune regulation and antigen presentation. These findings open new avenues for understanding SA and offer a foundation for future investigations into immune‐related pathways in skin diseases.

## 1 Introduction

Skin atrophy (SA) is a pathological condition characterized by the thinning of the skin, reduction in skin layers, and weakening of tissue function. These hallmark features could make the skin fragile, dry, less elastic, more prone to damage, and wrinkled [1]. Common causes of SA include aging, long‐term use of glucocorticoids (which inhibit fibroblast activity), and certain skin conditions such as lupus erythematosus and psoriasis [2]. SA can also occur during the repair process following inflammation. Patients with SA often exhibit notable visible changes, including deepened wrinkles, skin laxity, and altered pigmentation, which can negatively impact their self‐esteem and social confidence [3, 4]. Additionally, chronic SA requires long‐term medical management, with frequent visits and medication use that may contribute to economic burden and reduced quality of life [5]. The primary goal of treatment is to slow disease progression and restore skin barrier function, but more effective interventions are lacking [6]. Consequently, we are considering whether identifying the genes sensitive to SA may offer deeper insights into managing this condition.

The major histocompatibility complex (MHC) on chromosome 6 is crucial to the human immune system's function and plays a significant role in the onset and progression of various autoimmune skin diseases [7]. The MHC region contains many genes, especially human leukocyte antigen (HLA) genes, which are closely involved in regulating individual immune responses [8]. Studies have shown that genetic variations in the MHC region are strongly associated with the genetic susceptibility to multiple skin diseases, including psoriasis, atopic dermatitis, and lupus erythematosus [9, 10].

Summary‐data‐based Mendelian Randomization (SMR) is a method that utilizes summary‐level data to explore causal relationships between genetic variants, exposure factors, and disease outcomes [11]. This method is widely used in epidemiology and has been applied in numerous recent studies [12, 13]. The GTEx project, initiated by the National Institutes of Health (NIH), aims to collect tissue samples from donors to measure gene expression levels and genotype data across different tissues [14]. In this study, we selected three skin‐related datasets from the GTEx project for eQTL data as exposure, and SA GWAS data from the FinnGen database as the outcome for SMR analysis. Further, we conducted functional enrichment, colocalization, and drug enrichment analyses on the core genes (intersection genes) to explore their functions and druggability.

## 2 Methods

### 2.1 Study Design and Data Source

**FIGURE 1:**
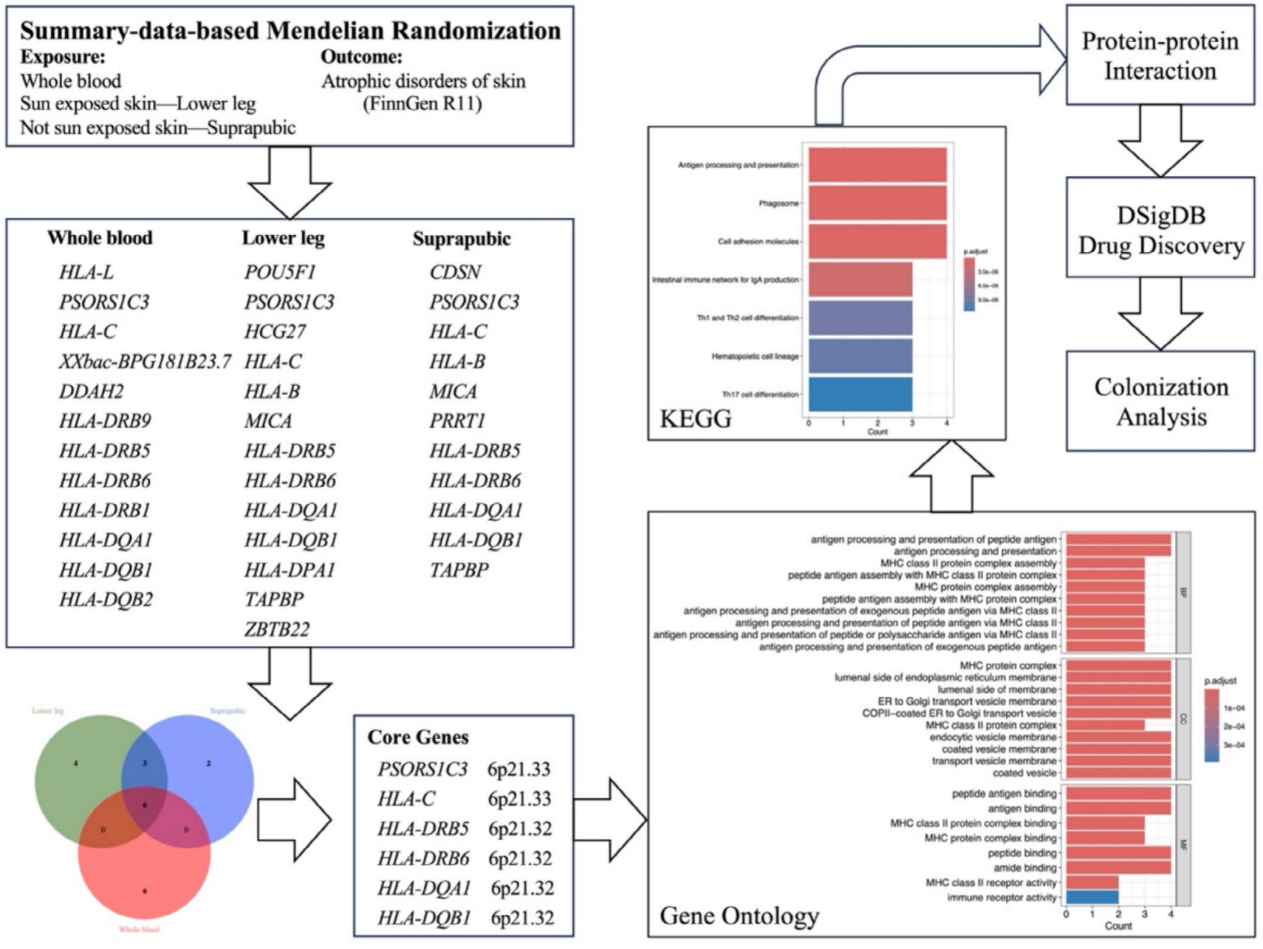
Overall design of the study.

In this study, we employed SMR analysis to evaluate the causal relationship between whole‐transcriptome gene expression (exposure) and SA (outcome) (Figure 1). The summary statistics from expression quantitative trait loci (eQTL) studies provided insights into the impact of genetic variants on gene expression levels. Since expression levels vary by tissue type, eQTL studies are generally tissue‐specific [15]. In each tissue, probes are used to measure gene expression, and cis‐eQTLs are defined as any SNP located within 1 Mb of the gene probe that is significantly associated with gene expression at a threshold of *P*eQTL < 5E−8. Cis‐eQTLs were obtained from three distinct tissues in the GTEx project version 8: whole blood, lower leg (sun‐exposed), and suprapubic (not sun‐exposed). The outcome data were derived from the European populations GWAS results of the FinnGen database [16].

We subsequently conducted SMR analyses using eQTL data from these three tissues separately and identified the core genes by intersecting the positive results across all tissues.

We performed in‐depth analyses of the core genes, focusing particularly on their biological function and druggability. Functional analysis included Gene Ontology (GO), Kyoto Encyclopedia of Genes and Genomes (KEGG) pathway analysis, and PPI network analysis. We then conducted drug enrichment analysis on these core genes to assess their potential as drug targets, evaluating the biological relevance and pharmacological value. Understanding the interaction between proteins and drugs is crucial for determining whether target proteins can serve as viable drug targets. In this study, we utilized the Drug Signature Database (DSigDB). Finally, we performed Bayesian colocalization analysis on the core genes to determine whether the two phenotypes in a given region share the same causal variant. All *P*‐values were adjusted using the Benjamini‐Hochberg method to control the false discovery rate (FDR) at *α* = 0.05 [17]. Analyses were conducted using the ‘TwoSampleMR’ package in R (version 4.2.3, http://www.R‐project.org, The R Foundation).

### 2.2 SMR Analysis and HEIDI Test

The SMR analysis (software version 1.3.1), developed by Yang's lab, is a method based on summary‐level genetic data that detects the causal relationship between gene expression levels and specific diseases, helping to identify potential pathogenic genes [18]. Due to the random nature of genetic variants, SMR effectively reduces confounding factors and reverse causality, which are common challenges in traditional observational studies. A top‐associated cis‐QTL was identified within a 1 Mb range centered around the respective gene, meeting the significance threshold of 5E‐8. To distinguish between pleiotropy and linkage, we employed the HEIDI test [19]. Variants with *p*‐HEIDI < 0.05 were considered pleiotropic and thus excluded from further analysis. We conducted three separate SMR analyses in total. Ultimately, core genes were identified by taking the intersection of the genes that met the criteria of an adjusted *p*‐value < 0.05 and *p*‐HEIDI > 0.05 across all three analyses. These genes represent the positive results common to all analyses.

### 2.3 Enrichment Analysis

To investigate the functional characteristics and biological significance of the identified potential therapeutic targets, we performed GO and KEGG pathway analyses using the R package ClusterProfiler [20]. The GO analysis encompasses three categories: biological process (BP), molecular function (MF), and cellular component (CC), providing a comprehensive view of the roles these genes may play in various biological contexts. KEGG provides information about metabolic and signal transduction pathways. These analyses were conducted to identify enriched pathways and processes linked to the identified proteins, helping to uncover the molecular mechanisms and pathways potentially involved in disease pathology and therapeutic response. During both analyses, we set adjusted *p*‐value < 0.05 as the threshold.

### 2.4 PPI Network Construction

STRING (Search Tool for the Retrieval of Interacting Genes/Proteins) is a comprehensive database and network resource designed to predict and analyze PPIs [21]. In this system, each protein is represented as a node, and the edges between nodes indicate interactions between proteins. Each interaction is assigned a confidence score based on its source and supporting evidence, where a higher score reflects a more reliable interaction. Researchers can utilize these scores to filter and prioritize key protein interactions. In our analysis, we have set the minimum required interaction score to 0.9, the highest confidence.

### 2.5 DSigDB Enrichment for Potential Drug Candidate

To explore potential drug candidates that may target the identified core genes, we conducted a DSigDB enrichment analysis [22]. DSigDB is a curated database of drug signatures containing gene expression profiles of various compounds under different experimental conditions. Enrichment results were ranked based on their *p*‐values, and we focused on the top drug signatures with an adjusted *p*‐value < 0.05.

### 2.6 Colocalization Analysis

The primary goal of colocalization analysis is to determine whether the genetic variants that regulate the exposure factors (core genes) and the outcome (SA) are the same. We conducted colocalization analysis using the coloc R package [23]. Within the colocalization analysis, five different posterior probabilities hypotheses were reported: (1) no causal variants for either trait (H_0_); (2) causal variants only for plasma proteins expression (H_1_); (3) causal variants only for disease risk (H_2_); (4) distinct causal variants for both traits (H_3_); and (5) shared causal variants for both traits (H_4_). eQTLs with high‐support evidence of colocalization (*P*H_4_ > 0.8) were considered positive results.

## 3 Results

### 3.1 Core Genes Acquisition

**FIGURE 2:**
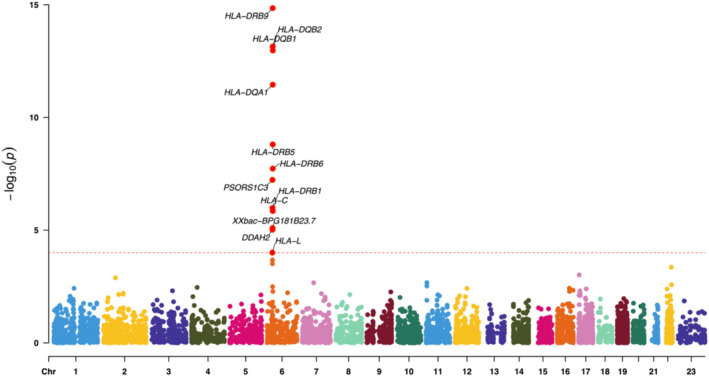
The Manhattan plot of SMR analysis using the whole blood eQTLs as the exposure.

**FIGURE 3:**
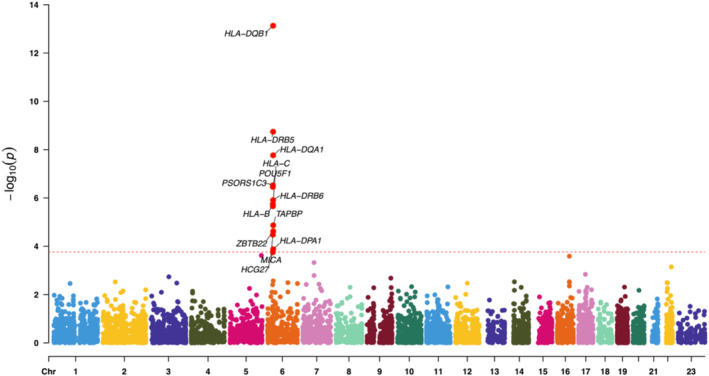
The Manhattan plot of SMR analysis using the lower leg eQTLs as the exposure.

**FIGURE 4:**
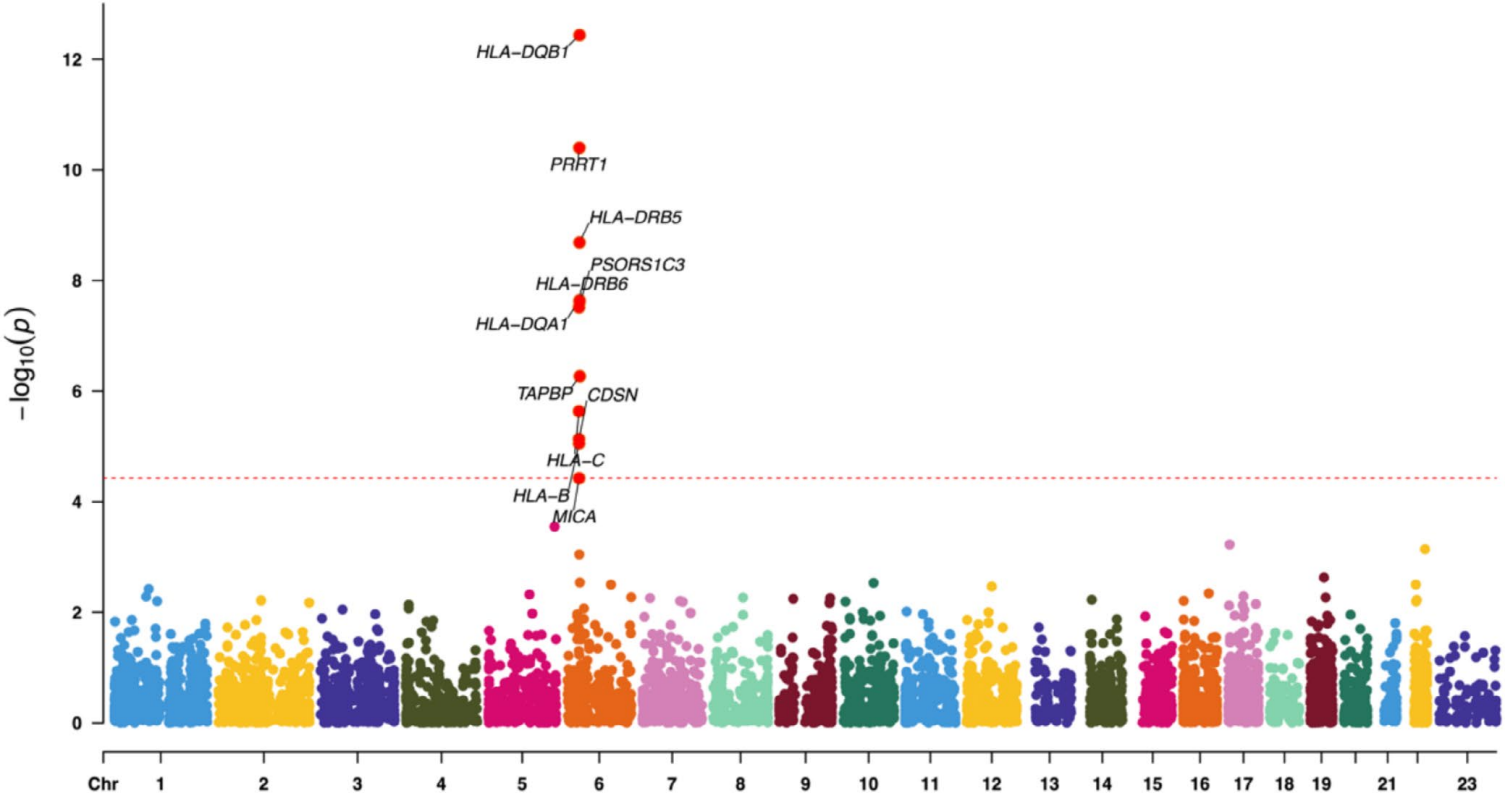
The Manhattan plot of SMR analysis using the suprapubic eQTLs as the exposure.

**FIGURE 5:**
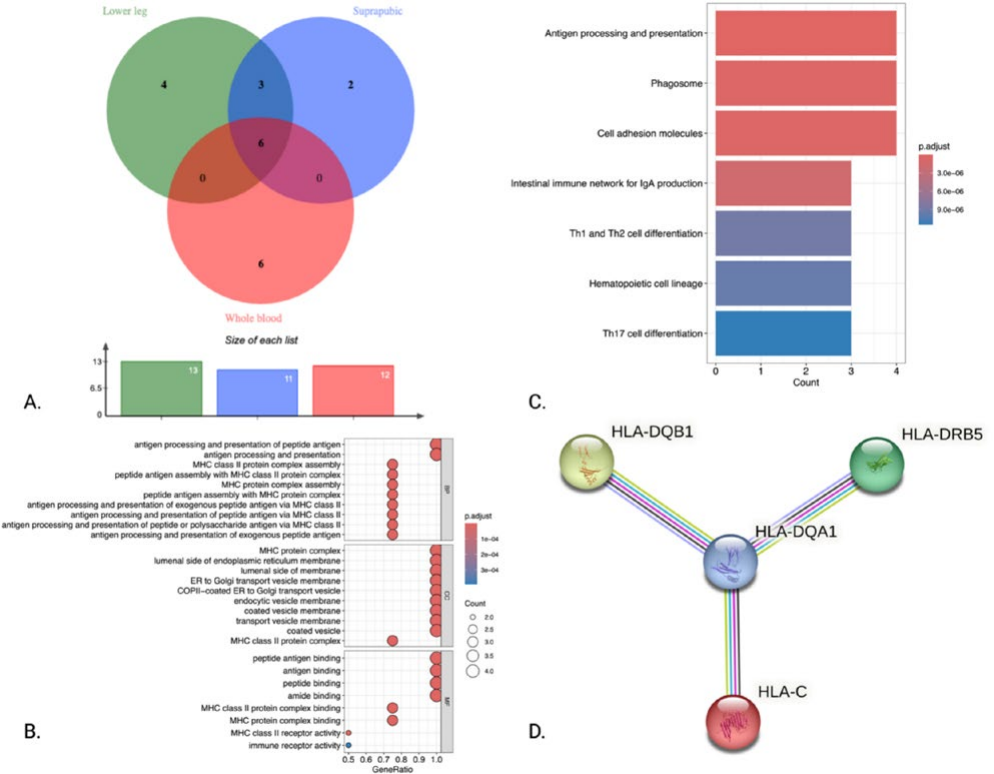
(A) The Venn diagram was used to identify the core genes by taking the intersection of gene sets. (B) GO analysis was performed to explore the BP, CC, and MF associated with the core genes. (C) KEGG pathway analysis was conducted to identify the key signaling pathways in which the core genes are involved. (D) A PPI network was constructed to visualize the interactions among the proteins encoded by the core genes.

In three rounds of SMR analysis, after applying strictly controlled threshold filtering, we consistently identified several susceptible genes. In the analysis using whole blood data as the exposure, 12 genes were identified, while analyses of lower leg and suprapubic data resulted in 13 and 11 genes, respectively (Tables S2–S4). Visualizing the results with a Manhattan plot, interestingly, we observed that all positive associations were located on chromosome 6 (Figures 2, 3, 4). We then took the intersection of the three sets of results. Fortunately, we identified six core genes: *PSORS1C3*, *HLA‐C*, *HLA‐DRB5*, *HLA‐DRB6*, *HLA‐DQA1*, and *HLA‐DQB1* (Figure 5A). Upon querying the NCBI database, we found that, remarkably, all of these genes are located at 6p21 (Table S1).

### 3.2 Functional Analysis of Core Genes

During the initial identification process using ClusterProfiler, we only retrieved information for four genes, with no identification of *PSORS1C3* or *HLA‐DRB6*. Notably, the significance in both GO and KEGG results was extremely high, and the enrichment ratio was also remarkable (Tables S5 and S6). The most significant BP was *antigen processing and presentation of peptide antigen*, with the remaining positive results predominantly centered around MHC protein. For the CC, aside from the most significant result of the MHC protein complex, the remaining positive associations were primarily linked to vesicles and membranes. The MF results also indicated that peptide antigen binding plays a key role (Figure 5B).

The top three significant results from the KEGG analysis were *Antigen processing and presentation*, *Phagosome*, and *Cell adhesion molecules* (Figure 5C). Additionally, during the construction of the PPI network using STRING, we again did not obtain data related to *PSORS1C3* or *HLA‐DRB6* (Table S7). Even after setting the confidence level to the highest, we still observed strong interaction relationships among the core genes (Figure 5D).

### 3.3 Candidate Drug Predication and Colonization

**FIGURE 6:**
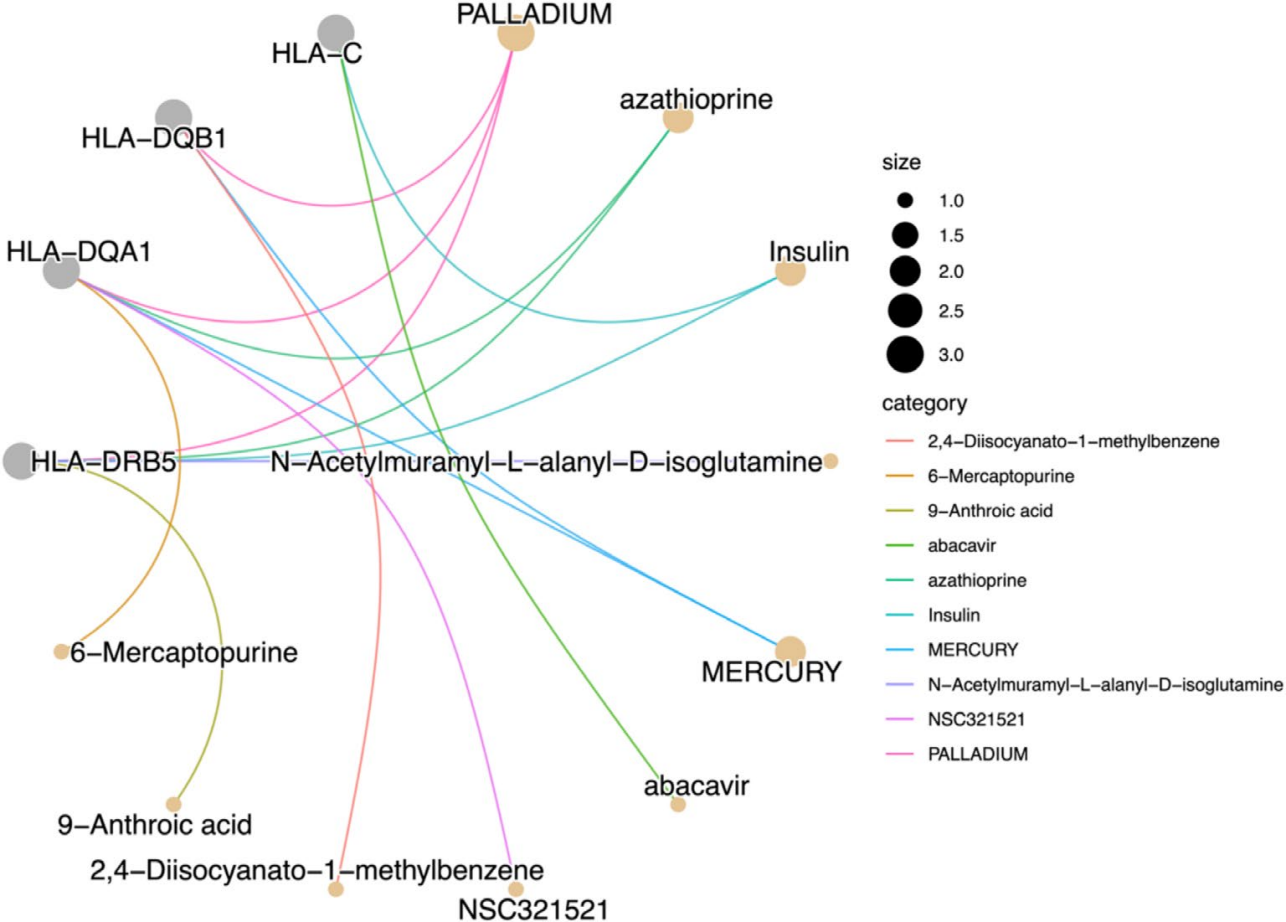
Drug enrichment analysis of the core genes was conducted using the DSigDB.

Unfortunately, the DSigDB database also did not provide data for *PSORS1C3* or HLA‐DRB6 (Table S8). However, the DSigDB enrichment analysis revealed significant drug‐gene interactions, predominantly involving immune‐related pathways (Figure 6). Palladium exhibited the strongest association, interacting with *HLA‐DRB5*, *HLA‐DQA1*, and *HLA‐DQB1*, suggesting its potential role in immune modulation. Other notable compounds included Azathioprine and Insulin, both of which interacted with key immune genes such as *HLA‐DRB5* and *HLA‐C*. Additionally, Mercury and N‐Acetylmuramyl‐L‐alanyl‐D‐isoglutamine showed significant interactions with *HLA‐DQA1* and *HLA‐DRB5*. Ultimately, the colocalization analysis for all the core genes did not yield any positive results.

## 4 Discussion

In this study, we identified six core genes located on 6p23 that are implicated in SA. Through subsequent functional gene set analyses, we obtained comprehensive results. Additionally, we explored the druggability of these genes.

Research on chromosome 6 and skin diseases has already established some foundational knowledge. Notably, the lack of expression of keratins K1 and K10 has been recognized as a valuable marker for the progression of skin tumors. These changes typically coincide with the transition of papillomas from a diploid to an aneuploid state, largely due to trisomies of chromosomes 6 and 7 [24]. Alexander Asamoah and colleagues reported a 6.5‐year‐old girl with a balanced translocation between the short arms of chromosomes 1 and 6, where one of the notable phenotypes was xerosis (dry skin) [25]. While these discoveries were made some time ago, there have been more recent advances as well. A case study from Japan revealed that a homozygous deletion of six genes, including *CDSN* on chromosome 6p21.3, is associated with generalized peeling skin disease [26]. Similarly, in a recent GWAS study on urticaria, the key pathogenic role of the HLA region on chromosome 6 was uncovered [27].

*PSORS1C3*, *HLA‐C*, *HLA‐DRB5*, *HLA‐DRB6*, *HLA‐DQA1*, and *HLA‐DQB1* are all located in the MHC region and play a pivotal role in the regulation of immune responses. Studies have shown that these genes participate in antigen presentation by encoding MHC class I and class II molecules, which are fundamental for the immune system's recognition and response to pathogens [28]. Importantly, these genes have been strongly linked to various autoimmune diseases, such as psoriasis, multiple sclerosis, and type 1 diabetes, suggesting their role in T‐cell‐mediated immune regulation. Our study is the first to report their crucial involvement in SA [29].

The shared function of these genes within the immune system is tied to their role in antigen presentation and T‐cell activation. MHC class I genes, such as *HLA‐C*, regulate the activation of cytotoxic T cells (CD8+ T cells) by presenting endogenous antigens, while MHC class II genes, such as *HLA‐DRB5* and *HLA‐DQA1*, activate helper T cells (CD4+ T cells) through exogenous antigen presentation [30]. Dysregulation or variation in these genes can lead to an immune system that erroneously attacks self‐tissues, resulting in autoimmune diseases [31]. This suggests that these genes may be pivotal in the mechanisms of immune dysregulation and abnormal immune responses, particularly in the recognition and regulation of self‐antigens by T cells.

In addition to these core genes, it is worth discussing whether the SMR analysis results of skin tissues under prolonged sunlight exposure showed any significant differences. We identified five positive genes: *CDSN* and *PRRT1* in lower leg skin, and *HCG27*, *HLA‐DPA1*, and *ZBTB22* in suprapubic skin [32, 33]. According to the SMR analysis, all of these genes are sensitive to SA, although the influence of sunlight on these differential expressions was not a focus of our study, it is still worth providing this result as a reference for other researchers.

In our exploration of druggability, we discovered the unique effects of palladium. Palladium is a metal commonly used in jewelry and dental instruments but has been shown to potentially cause metal allergies. Therefore, its application to the skin requires novel delivery systems. A mixture of platinum and palladium nanoparticles exhibits both superoxide dismutase and catalase‐like activities [34]. Topical application of this mixture improved SA caused by chronic oxidative damage in aged mouse models. Unlike the severe reactions induced by palladium chloride, this treatment method does not provoke skin inflammation [35]. Additionally, we attempted molecular docking studies between Azathioprine and insulin and our core genes. Unfortunately, we were unable to obtain the complete protein structure for the core genes, which limited further docking analysis.

However, there are two major limitations in our study. First, none of the analytical tools yielded relevant data for *PSORS1C3* and *HLA‐DRB6*. Our database search revealed that research on *PSORS1C3*, as its name suggests, primarily focuses on its involvement in psoriasis, while studies on *HLA‐DRB6* have stagnated since the 1990s [36, 37, 38]. The role of these genes in SA remains unclear and warrants further investigation. Second, no positive results were obtained from the colocalization analysis, and this was not due to a high threshold setting. Current mainstream views suggest that for two phenotypes to colocalize, it indicates that their causal relationship is genetically driven. However, during the HEIDI test in the SMR analysis, the top SNPs that we indirectly filtered out also represent a form of confirmation for genetic association. Unfortunately, we currently find it difficult to explain the conflicting conclusions between these analyses. On the positive side, this suggests that there is substantial room for further research [39].

## 5 Conclusion

This study highlights the involvement of six core genes within the MHC region on chromosome 6 in the development of SA, emphasizing their roles in immune regulation and antigen presentation. Functional and drug enrichment analyses provided insights into potential therapeutic targets, with palladium nanoparticles showing promising effects in SA models. However, data limitations, particularly for *PSORS1C3* and *HLA‐DRB6*, and conflicting results in the colocalization analysis underline the necessity for additional research to fully elucidate the genetic mechanisms underlying skin atrophy and to identify viable therapeutic strategies. These findings open new avenues for understanding SA and offer a foundation for future investigations into immune‐related pathways in skin diseases.

## Author Contributions

C.Z. conceptualized and drafted the manuscript. Z.F. and Y.S. provided information for the work. R.Z. was responsible for the language check. W.L. revised the manuscript. All authors read and approved the final manuscript.

## Ethics Statement

Since this study used publicly available data, no ethical approval was required.

## Conflicts of Interest

The authors declare no conflicts of interest.

## Supporting information


Data S1.

## Data Availability

The data that support the findings of this study are available on request from the corresponding author. The data are not publicly available due to privacy or ethical restrictions.
