# Supplementary material for: The Role of the Major Histocompatibility Complex Region on Chromosome 6 in Skin Atrophy: A Mendelian Randomization Study
Source: J Cosmet Dermatol. 2025 Mar 18;24(3):e70040. doi: 10.1111/jocd.70040 (PMC11915082; doi:10.1111/jocd.70040)
Supplement: Supplementary file 1 — Data S1. [file JOCD-24-e70040-s001.docx]

| **Gene** | **Genome** | **Probe ID** | **Exposure** | **Top SNP** | **b_GWAS** |
| --- | --- | --- | --- | --- | --- |
| PSORS1C3 | 6p21.33 | ENSG00000204528 | Whole blood | rs9380220 | 0.130852 |
|  |  |  | Lower leg | rs28749548 | 0.126845 |
|  |  |  | Suprapubic | rs9380221 | 0.131651 |
| HLA-C | 6p21.33 | ENSG00000204525 | Whole blood | rs2524105 | 0.10257 |
|  |  |  | Lower leg | rs9264396 | 0.0996808 |
|  |  |  | Suprapubic | rs9264396 | 0.0996808 |
| HLA-DRB5 | 6p21.32 | ENSG00000198502 | Whole blood | rs35083819 | 0.18384 |
|  |  |  | Lower leg | rs35083819 | 0.18384 |
|  |  |  | Suprapubic | rs35083819 | 0.18384 |
| HLA-DRB6 | 6p21.32 | ENSG00000229391 | Whole blood | rs28366333 | 0.134895 |
|  |  |  | Lower leg | rs9271434 | 0.105331 |
|  |  |  | Suprapubic | rs28366333 | 0.134895 |
| HLA-DQA1 | 6p21.32 | ENSG00000196735 | Whole blood | rs17843580 | 0.152885 |
|  |  |  | Lower leg | rs9271498 | 0.13567 |
|  |  |  | Suprapubic | rs9272998 | 0.135937 |
| HLA-DQB1 | 6p21.32 | ENSG00000179344 | Whole blood | rs3134993 | 0.15307 |
|  |  |  | Lower leg | rs3828811 | 0.152664 |
|  |  |  | Suprapubic | rs3828811 | 0.152664 |

Supplementary table 1. Core genes info.

|  |  | |  |  | |  |  | |  | |  | |  | |  | | | |  | |  | |  | |  | |  | |  | | |  |  |  |  |  |
| --- | --- | --- | --- | --- | --- | --- | --- | --- | --- | --- | --- | --- | --- | --- | --- | --- | --- | --- | --- | --- | --- | --- | --- | --- | --- | --- | --- | --- | --- | --- | --- | --- | --- | --- | --- | --- |
| probeID | | ProbeChr | | | Gene | | | Probe_bp | | topSNP | | topSNP_bp | | A1 | | A2 | Freq | b_GWAS | | se_GWAS | | p_GWAS | | b_eQTL | | se_eQTL | | p_eQTL | | b_SMR | se_SMR | | p_SMR | p_HEIDI | nsnp_HEIDI | qvalue |
| ENSG00000243753 | | 6 | | | HLA-L | | | 30227361 | | rs6904845 | | 31224423 | | C | | T | 0.751491 | 0.103269 | | 0.025239 | | 4.28341e-5 | | 0.705186 | | 0.055791 | | 1.273984e-36 | | 0.146442 | 0.0376191 | | 9.910833e-5 | 0.06807232 | 20 | 0.02617497 |
| ENSG00000204528 | | 6 | | | PSORS1C3 | | | 31141512 | | rs9380220 | | 31146584 | | T | | G | 0.200795 | 0.130852 | | 0.0227144 | | 8.37471e-9 | | 0.634634 | | 0.0394818 | | 3.876802e-58 | | 0.206185 | 0.0380205 | | 5.860721e-8 | 0.8014806 | 20 | 4.864651873872483e-5 |
| ENSG00000204525 | | 6 | | | HLA-C | | | 31236526 | | rs2524105 | | 31232910 | | C | | G | 0.409543 | 0.10257 | | 0.0201702 | | 3.67232e-7 | | -0.448997 | | 0.0255675 | | 4.881879e-69 | | -0.228443 | 0.0467683 | | 1.036613e-6 | 0.3584837 | 20 | 6.02303532458106e-4 |
| ENSG00000272221 | | 6 | | | XXbac-BPG181B23.7 | | | 31362066 | | rs75098758 | | 31361004 | | C | | T | 0.294235 | -0.0994111 | | 0.0221146 | | 6.94848e-6 | | -0.859369 | | 0.0333588 | | 2.40267e-146 | | 0.115679 | 0.0261224 | | 9.495402e-6 | 0.05996036 | 20 | 0.0034482 |
| ENSG00000213722 | | 6 | | | DDAH2 | | | 31694815 | | rs3130487 | | 31723389 | | T | | C | 0.894632 | 0.211978 | | 0.0310068 | | 8.11521e-12 | | -0.134431 | | 0.02282 | | 3.840253e-9 | | -1.57685 | 0.353341 | | 8.093696e-6 | 0.06396289 | 20 | 0.0034482 |
| ENSG00000196301 | | 6 | | | HLA-DRB9 | | | 32427598 | | rs9271418 | | 32587832 | | G | | A | 0.66501 | 0.193502 | | 0.0204368 | | 2.84381e-21 | | 0.654765 | | 0.044074 | | 6.358015e-50 | | 0.295529 | 0.0370127 | | 1.410643e-15 | 0.526499 | 20 | 8.196262847729094e-12 |
| ENSG00000198502 | | 6 | | | HLA-DRB5 | | | 32485120 | | rs35083819 | | 32526526 | | G | | A | 0.852883 | 0.18384 | | 0.0294981 | | 4.59759e-10 | | -0.950592 | | 0.0391641 | | 3.871846e-130 | | -0.193395 | 0.0320379 | | 1.5754e-9 | 0.08792873 | 20 | 1.8307101783105172e-6 |
| ENSG00000229391 | | 6 | | | HLA-DRB6 | | | 32520490 | | rs28366333 | | 32561887 | | C | | G | 0.236581 | 0.134895 | | 0.0230286 | | 4.69289e-9 | | 0.829607 | | 0.041248 | | 5.7157629999999995e-90 | | 0.162601 | 0.0289118 | | 1.865376e-8 | 0.9999436 | 20 | 1.8063998717659852e-5 |
| ENSG00000196126 | | 6 | | | HLA-DRB1 | | | 32546546 | | rs113568276 | | 32513127 | | A | | G | 0.257455 | 0.125514 | | 0.0239778 | | 1.65333e-7 | | -0.235707 | | 0.0189896 | | 2.237116e-35 | | -0.5325 | 0.110403 | | 1.412512e-6 | 0.1387501 | 20 | 7.080018479094466e-4 |
| ENSG00000196735 | | 6 | | | HLA-DQA1 | | | 32595956 | | rs17843580 | | 32615551 | | G | | A | 0.506958 | 0.152885 | | 0.0201369 | | 3.14413e-14 | | -0.356525 | | 0.0205582 | | 2.259293e-67 | | -0.42882 | 0.0616565 | | 3.526195e-12 | 1 | 20 | 5.122065092363571e-9 |
| ENSG00000179344 | | 6 | | | HLA-DQB1 | | | 32627244 | | rs3134993 | | 32637778 | | A | | G | 0.592445 | 0.15307 | | 0.0201441 | | 2.99089e-14 | | -0.775797 | | 0.0178434 | | 0 | | -0.197307 | 0.0263593 | | 7.138882e-14 | 1 | 20 | 2.0658143936898005e-10 |
| ENSG00000232629 | | 6 | | | HLA-DQB2 | | | 32723875 | | rs9274660 | | 32636434 | | G | | A | 0.595427 | 0.152335 | | 0.0201349 | | 3.85745e-14 | | 0.972685 | | 0.0244622 | | 0 | | 0.156613 | 0.0210717 | | 1.06663e-13 | 1 | 20 | 2.0658143936898005e-10 |

Supplementary table 2. Results of whole blood.

| **Skin_Not_Sun_Exposed_Suprapubic** | | |  |  |  |  |  |  |  | | |  |  |  |  |  |  |  |  |  |  |  |  |  |
| --- | --- | --- | --- | --- | --- | --- | --- | --- | --- | --- | --- | --- | --- | --- | --- | --- | --- | --- | --- | --- | --- | --- | --- | --- |
| probeID | ProbeChr | Gene | | | Probe_bp | topSNP | topSNP_bp | A1 | A2 | Freq | b_GWAS | | | se_GWAS | p_GWAS | b_eQTL | se_eQTL | p_eQTL | b_SMR | se_SMR | p_SMR | p_HEIDI | nsnp_HEIDI | qvalue |
| ENSG00000204539 | 6 | CDSN | | | 31082867 | rs3094198 | 31093327 | G | A | 0.450298 | 0.105208 | | | 0.0202304 | 1.98783e-7 | 0.238074 | 0.0269515 | 1.015226e-18 | 0.441913 | 0.098608 | 7.411557e-6 | 0.1714111 | 20 | 0.00451337 |
| ENSG00000204528 | 6 | PSORS1C3 | | | 31141512 | rs9380221 | 31150873 | C | T | 0.198807 | 0.131651 | | | 0.0227161 | 6.81224e-9 | 1.17714 | 0.0628078 | 2.2529049999999997e-78 | 0.11184 | 0.0201993 | 3.080158e-8 | 0.7669521 | 20 | 3.016431899984877e-5 |
| ENSG00000204525 | 6 | HLA-C | | | 31236526 | rs9264396 | 31229307 | T | C | 0.417495 | 0.0996808 | | | 0.0202702 | 8.76093e-7 | -0.667263 | 0.039283 | 1.0413959999999999e-64 | -0.149388 | 0.0316256 | 2.316711e-6 | 0.2416597 | 20 | 0.00158815 |
| ENSG00000234745 | 6 | HLA-B | | | 31321649 | rs56189237 | 31328518 | A | G | 0.146123 | 0.191671 | | | 0.0346206 | 3.0888e-8 | 0.334356 | 0.0448878 | 9.425993e-14 | 0.573254 | 0.129013 | 8.855017e-6 | 0.05673814 | 17 | 0.00466944 |
| ENSG00000204520 | 6 | MICA | | | 31371356 | rs1063631 | 31378387 | C | T | 0.314115 | -0.0912382 | | | 0.0214092 | 2.02932e-5 | -0.499124 | 0.0306976 | 1.915996e-59 | 0.182797 | 0.0443424 | 3.749661e-5 | 0.187973 | 20 | 0.014627824590438258 |
| ENSG00000204314 | 6 | PRRT1 | | | 32116136 | rs1800684 | 32151994 | T | A | 0.893638 | 0.261287 | | | 0.0316109 | 1.38867e-16 | 0.588827 | 0.0536002 | 4.485328e-28 | 0.443742 | 0.0671836 | 3.977731e-11 | 0.05453658 | 20 | 1.3634021817340412e-7 |
| ENSG00000198502 | 6 | HLA-DRB5 | | | 32485120 | rs35083819 | 32526526 | G | A | 0.852883 | 0.18384 | | | 0.0294981 | 4.59759e-10 | -1.34449 | 0.0615155 | 6.800856999999999e-106 | -0.136736 | 0.0228145 | 2.055422e-9 | 0.2232167 | 20 | 4.696759432591339e-6 |
| ENSG00000229391 | 6 | HLA-DRB6 | | | 32520490 | rs28366333 | 32561887 | C | G | 0.236581 | 0.134895 | | | 0.0230286 | 4.69289e-9 | 1.02992 | 0.0564315 | 2.03893e-74 | 0.130976 | 0.023483 | 2.440288e-8 | 0.999999 | 20 | 3.016431899984877e-5 |
| ENSG00000196735 | 6 | HLA-DQA1 | | | 32595956 | rs9272998 | 32611474 | A | G | 0.262425 | 0.135937 | | | 0.0228395 | 2.65118e-9 | -0.670738 | 0.0412821 | 2.320823e-59 | -0.202668 | 0.0362641 | 2.288209e-8 | 1 | 20 | 3.016431899984877e-5 |
| ENSG00000179344 | 6 | HLA-DQB1 | | | 32627244 | rs3828811 | 32636221 | G | T | 0.589463 | 0.152664 | | | 0.0201442 | 3.49462e-14 | -0.968768 | 0.0378306 | 1.2404899999999998e-144 | -0.157586 | 0.0216851 | 3.675473e-13 | 1 | 20 | 2.5196012033516405e-9 |
| ENSG00000231925 | 6 | TAPBP | | | 33267471 | rs3130259 | 33268737 | A | C | 0.293241 | 0.1394 | | | 0.020617 | 1.36647e-11 | -0.210483 | 0.0281952 | 8.316677e-14 | -0.662286 | 0.132155 | 5.40266e-7 | 0.56149 | 14 | 4.115131315464484e-4 |

Supplementary table 3. Results of suprapubic.

| **Skin_Sun_Exposed_Lower_leg** | | |  |  |  |  |  |  |  |  |  |  |  |  |  |  |  |  |  |  |
| --- | --- | --- | --- | --- | --- | --- | --- | --- | --- | --- | --- | --- | --- | --- | --- | --- | --- | --- | --- | --- |
| probeID | ProbeChr | Gene | Probe_bp | topSNP | topSNP_bp | A1 | A2 | Freq | b_GWAS | se_GWAS | p_GWAS | b_eQTL | se_eQTL | p_eQTL | b_SMR | se_SMR | p_SMR | p_HEIDI | nsnp_HEIDI | qvalue |
| ENSG00000204531 | 6 | POU5F1 | 31132119 | rs28749548 | 31150914 | A | T | 0.180915 | 0.126845 | 0.0238801 | 1.0859e-7 | 0.955689 | 0.0529813 | 9.759292e-73 | 0.132726 | 0.0260482 | 3.479748e-7 | 0.0904882 | 20 | 4.440505904092148e-4 |
| ENSG00000204528 | 6 | PSORS1C3 | 31141512 | rs28749548 | 31150914 | A | T | 0.180915 | 0.126845 | 0.0238801 | 1.0859e-7 | 1.16981 | 0.0602265 | 4.884511e-84 | 0.108432 | 0.0211632 | 2.997373e-7 | 0.5738912 | 20 | 4.440505904092148e-4 |
| ENSG00000206344 | 6 | HCG27 | 31165537 | rs7764413 | 31242082 | C | T | 0.595427 | 0.0830203 | 0.0208654 | 6.92484e-5 | 0.459441 | 0.0404294 | 6.316702e-30 | 0.180698 | 0.048118 | 1.731185e-4 | 0.6110577 | 20 | 0.04909255 |
| ENSG00000204525 | 6 | HLA-C | 31236526 | rs9264396 | 31229307 | T | C | 0.417495 | 0.0996808 | 0.0202702 | 8.76093e-7 | -0.619049 | 0.035315 | 8.556012000000001e-69 | -0.161022 | 0.0340082 | 2.19254e-6 | 0.1952314 | 20 | 0.00167874 |
| ENSG00000234745 | 6 | HLA-B | 31321649 | rs56189237 | 31328518 | A | G | 0.146123 | 0.191671 | 0.0346206 | 3.0888e-8 | 0.414531 | 0.0438482 | 3.268091e-21 | 0.46238 | 0.0967849 | 1.775757e-6 | 0.1621005 | 20 | 0.0015107 |
| ENSG00000204520 | 6 | MICA | 31371356 | rs1063631 | 31378387 | C | T | 0.314115 | -0.0912382 | 0.0214092 | 2.02932e-5 | -0.632253 | 0.0345606 | 9.227520000000001e-75 | 0.144306 | 0.0347684 | 3.317409e-5 | 0.1997152 | 20 | 0.012700035391173519 |
| ENSG00000198502 | 6 | HLA-DRB5 | 32485120 | rs35083819 | 32526526 | G | A | 0.852883 | 0.18384 | 0.0294981 | 4.59759e-10 | -1.41363 | 0.0616693 | 2.755992e-116 | -0.130048 | 0.0216244 | 1.810516e-9 | 0.08805279 | 20 | 4.62079839543168e-6 |
| ENSG00000229391 | 6 | HLA-DRB6 | 32520490 | rs9271434 | 32588058 | A | G | 0.333996 | 0.105331 | 0.0210687 | 5.75016e-7 | 0.928769 | 0.046542 | 1.342925e-88 | 0.113409 | 0.0233856 | 1.237519e-6 | 0.09834987 | 20 | 0.0011844 |
| ENSG00000196735 | 6 | HLA-DQA1 | 32595956 | rs9271498 | 32589282 | A | G | 0.293241 | 0.13567 | 0.0228716 | 2.99599e-9 | -0.722354 | 0.03981 | 1.405684e-73 | -0.187817 | 0.0333116 | 1.718586e-8 | 1 | 20 | 3.2896310076290475e-5 |
| ENSG00000179344 | 6 | HLA-DQB1 | 32627244 | rs3828811 | 32636221 | G | T | 0.589463 | 0.152664 | 0.0201442 | 3.49462e-14 | -1.08481 | 0.0232096 | 0 | -0.140729 | 0.0188119 | 7.383876e-14 | 1 | 20 | 5.653537837754512e-10 |
| ENSG00000231389 | 6 | HLA-DPA1 | 33032346 | rs6899851 | 33034113 | C | T | 0.186879 | -0.131684 | 0.033313 | 7.71969e-5 | -0.532684 | 0.0356831 | 2.160463e-50 | 0.247208 | 0.0646934 | 1.327857e-4 | 0.08347649 | 20 | 0.04066747 |
| ENSG00000231925 | 6 | TAPBP | 33267471 | rs3106189 | 33282002 | T | C | 0.497018 | 0.110118 | 0.0200739 | 4.12022e-8 | -0.154787 | 0.0216725 | 9.192967e-13 | -0.711416 | 0.163526 | 1.358413e-5 | 0.1554605 | 14 | 0.00729748 |
| ENSG00000236104 | 6 | ZBTB22 | 33282183 | rs1061783 | 33282628 | T | C | 0.506958 | 0.109122 | 0.020084 | 5.53235e-8 | 0.122389 | 0.0182193 | 1.848325e-11 | 0.8916 | 0.211057 | 2.395355e-5 | 0.2267273 | 16 | 0.010217803818763232 |

Supplementary table 4. Results of lower leg.

| ONTOLOGY | ID | Description | GeneRatio | BgRatio | pvalue | p.adjust | qvalue | geneID | Count |
| --- | --- | --- | --- | --- | --- | --- | --- | --- | --- |
| BP | GO:0048002 | antigen processing and presentation of peptide antigen | 100% | 72/18888 | 1.94E-10 | 1.20E-08 | 2.04E-09 | HLA-C/HLA-DRB5/HLA-DQA1/HLA-DQB1 | 4 |
| BP | GO:0019882 | antigen processing and presentation | 100% | 117/18888 | 1.40E-09 | 3.09E-08 | 5.25E-09 | HLA-C/HLA-DRB5/HLA-DQA1/HLA-DQB1 | 4 |
| BP | GO:0002399 | MHC class II protein complex assembly | 75% | 16/18888 | 1.99E-09 | 3.09E-08 | 5.25E-09 | HLA-DRB5/HLA-DQA1/HLA-DQB1 | 3 |
| BP | GO:0002503 | peptide antigen assembly with MHC class II protein complex | 75% | 16/18888 | 1.99E-09 | 3.09E-08 | 5.25E-09 | HLA-DRB5/HLA-DQA1/HLA-DQB1 | 3 |
| BP | GO:0002396 | MHC protein complex assembly | 75% | 21/18888 | 4.73E-09 | 4.89E-08 | 8.31E-09 | HLA-DRB5/HLA-DQA1/HLA-DQB1 | 3 |
| BP | GO:0002501 | peptide antigen assembly with MHC protein complex | 75% | 21/18888 | 4.73E-09 | 4.89E-08 | 8.31E-09 | HLA-DRB5/HLA-DQA1/HLA-DQB1 | 3 |
| BP | GO:0019886 | antigen processing and presentation of exogenous peptide antigen via MHC class II | 75% | 31/18888 | 1.60E-08 | 1.42E-07 | 2.41E-08 | HLA-DRB5/HLA-DQA1/HLA-DQB1 | 3 |
| BP | GO:0002495 | antigen processing and presentation of peptide antigen via MHC class II | 75% | 35/18888 | 2.33E-08 | 1.80E-07 | 3.06E-08 | HLA-DRB5/HLA-DQA1/HLA-DQB1 | 3 |
| BP | GO:0002504 | antigen processing and presentation of peptide or polysaccharide antigen via MHC class II | 75% | 37/18888 | 2.76E-08 | 1.90E-07 | 3.23E-08 | HLA-DRB5/HLA-DQA1/HLA-DQB1 | 3 |
| BP | GO:0002478 | antigen processing and presentation of exogenous peptide antigen | 75% | 41/18888 | 3.79E-08 | 2.35E-07 | 3.99E-08 | HLA-DRB5/HLA-DQA1/HLA-DQB1 | 3 |
| BP | GO:0019884 | antigen processing and presentation of exogenous antigen | 75% | 50/18888 | 6.97E-08 | 3.93E-07 | 6.67E-08 | HLA-DRB5/HLA-DQA1/HLA-DQB1 | 3 |
| BP | GO:0050870 | positive regulation of T cell activation | 75% | 253/18888 | 9.41E-06 | 4.86E-05 | 8.25E-06 | HLA-DRB5/HLA-DQA1/HLA-DQB1 | 3 |
| BP | GO:1903039 | positive regulation of leukocyte cell-cell adhesion | 75% | 277/18888 | 1.23E-05 | 5.89E-05 | 1.00E-05 | HLA-DRB5/HLA-DQA1/HLA-DQB1 | 3 |
| BP | GO:0022409 | positive regulation of cell-cell adhesion | 75% | 325/18888 | 1.99E-05 | 8.47E-05 | 1.44E-05 | HLA-DRB5/HLA-DQA1/HLA-DQB1 | 3 |
| BP | GO:0051251 | positive regulation of lymphocyte activation | 75% | 328/18888 | 2.05E-05 | 8.47E-05 | 1.44E-05 | HLA-DRB5/HLA-DQA1/HLA-DQB1 | 3 |
| BP | GO:0002696 | positive regulation of leukocyte activation | 75% | 362/18888 | 2.75E-05 | 0.000106 | 1.79E-05 | HLA-DRB5/HLA-DQA1/HLA-DQB1 | 3 |
| BP | GO:0050867 | positive regulation of cell activation | 75% | 380/18888 | 3.18E-05 | 0.000106 | 1.79E-05 | HLA-DRB5/HLA-DQA1/HLA-DQB1 | 3 |
| BP | GO:0050863 | regulation of T cell activation | 75% | 382/18888 | 3.23E-05 | 0.000106 | 1.79E-05 | HLA-DRB5/HLA-DQA1/HLA-DQB1 | 3 |
| BP | GO:1903037 | regulation of leukocyte cell-cell adhesion | 75% | 382/18888 | 3.23E-05 | 0.000106 | 1.79E-05 | HLA-DRB5/HLA-DQA1/HLA-DQB1 | 3 |
| BP | GO:0007159 | leukocyte cell-cell adhesion | 75% | 419/18888 | 4.26E-05 | 0.000132 | 2.24E-05 | HLA-DRB5/HLA-DQA1/HLA-DQB1 | 3 |
| BP | GO:0045785 | positive regulation of cell adhesion | 75% | 485/18888 | 6.60E-05 | 0.000195 | 3.31E-05 | HLA-DRB5/HLA-DQA1/HLA-DQB1 | 3 |
| BP | GO:0022407 | regulation of cell-cell adhesion | 75% | 496/18888 | 7.06E-05 | 0.000199 | 3.38E-05 | HLA-DRB5/HLA-DQA1/HLA-DQB1 | 3 |
| BP | GO:0002486 | antigen processing and presentation of endogenous peptide antigen via MHC class I via ER pathway, TAP-independent | 25% | 16/18888 | 0.003384 | 0.008917 | 0.001514 | HLA-C | 1 |
| BP | GO:0002476 | antigen processing and presentation of endogenous peptide antigen via MHC class Ib | 25% | 17/18888 | 0.003596 | 0.008917 | 0.001514 | HLA-C | 1 |
| BP | GO:0002484 | antigen processing and presentation of endogenous peptide antigen via MHC class I via ER pathway | 25% | 17/18888 | 0.003596 | 0.008917 | 0.001514 | HLA-C | 1 |
| BP | GO:0002428 | antigen processing and presentation of peptide antigen via MHC class Ib | 25% | 18/18888 | 0.003807 | 0.009078 | 0.001541 | HLA-C | 1 |
| BP | GO:0019885 | antigen processing and presentation of endogenous peptide antigen via MHC class I | 25% | 23/18888 | 0.004862 | 0.011165 | 0.001896 | HLA-C | 1 |
| BP | GO:0002475 | antigen processing and presentation via MHC class Ib | 25% | 25/18888 | 0.005284 | 0.011297 | 0.001918 | HLA-C | 1 |
| BP | GO:0002483 | antigen processing and presentation of endogenous peptide antigen | 25% | 25/18888 | 0.005284 | 0.011297 | 0.001918 | HLA-C | 1 |
| BP | GO:0019883 | antigen processing and presentation of endogenous antigen | 25% | 32/18888 | 0.00676 | 0.013971 | 0.002372 | HLA-C | 1 |
| BP | GO:0002474 | antigen processing and presentation of peptide antigen via MHC class I | 25% | 37/18888 | 0.007813 | 0.015627 | 0.002653 | HLA-C | 1 |
| BP | GO:0001916 | positive regulation of T cell mediated cytotoxicity | 25% | 40/18888 | 0.008445 | 0.016362 | 0.002778 | HLA-C | 1 |
| BP | GO:0001914 | regulation of T cell mediated cytotoxicity | 25% | 50/18888 | 0.010548 | 0.019817 | 0.003364 | HLA-C | 1 |
| BP | GO:0001913 | T cell mediated cytotoxicity | 25% | 60/18888 | 0.012647 | 0.023062 | 0.003915 | HLA-C | 1 |
| BP | GO:0001912 | positive regulation of leukocyte mediated cytotoxicity | 25% | 69/18888 | 0.014534 | 0.02503 | 0.00425 | HLA-C | 1 |
| BP | GO:0002711 | positive regulation of T cell mediated immunity | 25% | 69/18888 | 0.014534 | 0.02503 | 0.00425 | HLA-C | 1 |
| BP | GO:0031343 | positive regulation of cell killing | 25% | 76/18888 | 0.015999 | 0.02681 | 0.004552 | HLA-C | 1 |
| BP | GO:0001910 | regulation of leukocyte mediated cytotoxicity | 25% | 96/18888 | 0.020177 | 0.032921 | 0.005589 | HLA-C | 1 |
| BP | GO:0002709 | regulation of T cell mediated immunity | 25% | 99/18888 | 0.020803 | 0.033072 | 0.005615 | HLA-C | 1 |
| BP | GO:0031341 | regulation of cell killing | 25% | 110/18888 | 0.023094 | 0.035796 | 0.006077 | HLA-C | 1 |
| BP | GO:0002824 | positive regulation of adaptive immune response based on somatic recombination of immune receptors built from immunoglobulin superfamily domains | 25% | 125/18888 | 0.026212 | 0.03869 | 0.006569 | HLA-C | 1 |
| BP | GO:0002708 | positive regulation of lymphocyte mediated immunity | 25% | 128/18888 | 0.026835 | 0.03869 | 0.006569 | HLA-C | 1 |
| BP | GO:0002456 | T cell mediated immunity | 25% | 129/18888 | 0.027042 | 0.03869 | 0.006569 | HLA-C | 1 |
| BP | GO:0002821 | positive regulation of adaptive immune response | 25% | 131/18888 | 0.027457 | 0.03869 | 0.006569 | HLA-C | 1 |
| BP | GO:0001909 | leukocyte mediated cytotoxicity | 25% | 144/18888 | 0.030151 | 0.041196 | 0.006994 | HLA-C | 1 |
| BP | GO:0050852 | T cell receptor signaling pathway | 25% | 146/18888 | 0.030565 | 0.041196 | 0.006994 | HLA-DQB1 | 1 |
| BP | GO:0002705 | positive regulation of leukocyte mediated immunity | 25% | 150/18888 | 0.031392 | 0.041411 | 0.007031 | HLA-C | 1 |
| CC | GO:0042611 | MHC protein complex | 100% | 25/19894 | 1.94E-12 | 5.82E-11 | 5.75E-12 | HLA-C/HLA-DRB5/HLA-DQA1/HLA-DQB1 | 4 |
| CC | GO:0098553 | lumenal side of endoplasmic reticulum membrane | 100% | 29/19894 | 3.64E-12 | 5.82E-11 | 5.75E-12 | HLA-C/HLA-DRB5/HLA-DQA1/HLA-DQB1 | 4 |
| CC | GO:0098576 | lumenal side of membrane | 100% | 39/19894 | 1.26E-11 | 1.34E-10 | 1.33E-11 | HLA-C/HLA-DRB5/HLA-DQA1/HLA-DQB1 | 4 |
| CC | GO:0012507 | ER to Golgi transport vesicle membrane | 100% | 64/19894 | 9.74E-11 | 7.79E-10 | 7.69E-11 | HLA-C/HLA-DRB5/HLA-DQA1/HLA-DQB1 | 4 |
| CC | GO:0030134 | COPII-coated ER to Golgi transport vesicle | 100% | 94/19894 | 4.67E-10 | 2.99E-09 | 2.95E-10 | HLA-C/HLA-DRB5/HLA-DQA1/HLA-DQB1 | 4 |
| CC | GO:0042613 | MHC class II protein complex | 75% | 17/19894 | 2.07E-09 | 1.11E-08 | 1.09E-09 | HLA-DRB5/HLA-DQA1/HLA-DQB1 | 3 |
| CC | GO:0030666 | endocytic vesicle membrane | 100% | 204/19894 | 1.07E-08 | 4.38E-08 | 4.32E-09 | HLA-C/HLA-DRB5/HLA-DQA1/HLA-DQB1 | 4 |
| CC | GO:0030662 | coated vesicle membrane | 100% | 205/19894 | 1.10E-08 | 4.38E-08 | 4.32E-09 | HLA-C/HLA-DRB5/HLA-DQA1/HLA-DQB1 | 4 |
| CC | GO:0030658 | transport vesicle membrane | 100% | 238/19894 | 2.00E-08 | 7.10E-08 | 7.01E-09 | HLA-C/HLA-DRB5/HLA-DQA1/HLA-DQB1 | 4 |
| CC | GO:0030135 | coated vesicle | 100% | 317/19894 | 6.33E-08 | 2.02E-07 | 2.00E-08 | HLA-C/HLA-DRB5/HLA-DQA1/HLA-DQB1 | 4 |
| CC | GO:0030139 | endocytic vesicle | 100% | 350/19894 | 9.42E-08 | 2.74E-07 | 2.70E-08 | HLA-C/HLA-DRB5/HLA-DQA1/HLA-DQB1 | 4 |
| CC | GO:0030669 | clathrin-coated endocytic vesicle membrane | 75% | 75/19894 | 2.05E-07 | 5.47E-07 | 5.40E-08 | HLA-DRB5/HLA-DQA1/HLA-DQB1 | 3 |
| CC | GO:0030133 | transport vesicle | 100% | 434/19894 | 2.23E-07 | 5.50E-07 | 5.43E-08 | HLA-C/HLA-DRB5/HLA-DQA1/HLA-DQB1 | 4 |
| CC | GO:0045334 | clathrin-coated endocytic vesicle | 75% | 93/19894 | 3.94E-07 | 9.01E-07 | 8.89E-08 | HLA-DRB5/HLA-DQA1/HLA-DQB1 | 3 |
| CC | GO:0032588 | trans-Golgi network membrane | 75% | 106/19894 | 5.86E-07 | 1.25E-06 | 1.23E-07 | HLA-DRB5/HLA-DQA1/HLA-DQB1 | 3 |
| CC | GO:0030665 | clathrin-coated vesicle membrane | 75% | 138/19894 | 1.30E-06 | 2.60E-06 | 2.57E-07 | HLA-DRB5/HLA-DQA1/HLA-DQB1 | 3 |
| CC | GO:0031902 | late endosome membrane | 75% | 174/19894 | 2.61E-06 | 4.92E-06 | 4.86E-07 | HLA-DRB5/HLA-DQA1/HLA-DQB1 | 3 |
| CC | GO:0030136 | clathrin-coated vesicle | 75% | 218/19894 | 5.15E-06 | 9.16E-06 | 9.03E-07 | HLA-DRB5/HLA-DQA1/HLA-DQB1 | 3 |
| CC | GO:0005802 | trans-Golgi network | 75% | 260/19894 | 8.74E-06 | 1.47E-05 | 1.45E-06 | HLA-DRB5/HLA-DQA1/HLA-DQB1 | 3 |
| CC | GO:0005770 | late endosome | 75% | 314/19894 | 1.54E-05 | 2.46E-05 | 2.43E-06 | HLA-DRB5/HLA-DQA1/HLA-DQB1 | 3 |
| CC | GO:0098791 | Golgi apparatus subcompartment | 75% | 382/19894 | 2.77E-05 | 4.22E-05 | 4.17E-06 | HLA-DRB5/HLA-DQA1/HLA-DQB1 | 3 |
| CC | GO:0005765 | lysosomal membrane | 75% | 443/19894 | 4.31E-05 | 6.00E-05 | 5.92E-06 | HLA-DRB5/HLA-DQA1/HLA-DQB1 | 3 |
| CC | GO:0098852 | lytic vacuole membrane | 75% | 443/19894 | 4.31E-05 | 6.00E-05 | 5.92E-06 | HLA-DRB5/HLA-DQA1/HLA-DQB1 | 3 |
| CC | GO:0005774 | vacuolar membrane | 75% | 487/19894 | 5.73E-05 | 7.63E-05 | 7.53E-06 | HLA-DRB5/HLA-DQA1/HLA-DQB1 | 3 |
| CC | GO:0030670 | phagocytic vesicle membrane | 25% | 81/19894 | 0.016188 | 0.020721 | 0.002045 | HLA-C | 1 |
| CC | GO:0055038 | recycling endosome membrane | 25% | 108/19894 | 0.021541 | 0.026511 | 0.002616 | HLA-C | 1 |
| CC | GO:0045335 | phagocytic vesicle | 25% | 142/19894 | 0.028249 | 0.033481 | 0.003304 | HLA-C | 1 |
| CC | GO:0031901 | early endosome membrane | 25% | 195/19894 | 0.038638 | 0.044158 | 0.004358 | HLA-C | 1 |
| CC | GO:0055037 | recycling endosome | 25% | 206/19894 | 0.040784 | 0.045003 | 0.004441 | HLA-C | 1 |
| MF | GO:0042605 | peptide antigen binding | 100% | 47/18522 | 3.64E-11 | 2.91E-10 | NA | HLA-C/HLA-DRB5/HLA-DQA1/HLA-DQB1 | 4 |
| MF | GO:0003823 | antigen binding | 100% | 177/18522 | 8.06E-09 | 2.94E-08 | NA | HLA-C/HLA-DRB5/HLA-DQA1/HLA-DQB1 | 4 |
| MF | GO:0023026 | MHC class II protein complex binding | 75% | 27/18522 | 1.10E-08 | 2.94E-08 | NA | HLA-DRB5/HLA-DQA1/HLA-DQB1 | 3 |
| MF | GO:0023023 | MHC protein complex binding | 75% | 37/18522 | 2.93E-08 | 5.86E-08 | NA | HLA-DRB5/HLA-DQA1/HLA-DQB1 | 3 |
| MF | GO:0042277 | peptide binding | 100% | 336/18522 | 1.06E-07 | 1.70E-07 | NA | HLA-C/HLA-DRB5/HLA-DQA1/HLA-DQB1 | 4 |
| MF | GO:0033218 | amide binding | 100% | 409/18522 | 2.34E-07 | 3.12E-07 | NA | HLA-C/HLA-DRB5/HLA-DQA1/HLA-DQB1 | 4 |
| MF | GO:0032395 | MHC class II receptor activity | 50% | 10/18522 | 1.57E-06 | 1.80E-06 | NA | HLA-DQA1/HLA-DQB1 | 2 |
| MF | GO:0140375 | immune receptor activity | 50% | 151/18522 | 0.000392 | 0.000392 | NA | HLA-DQA1/HLA-DQB1 | 2 |

Supplementary table 5. Results of GO analysis.

| category | subcategory | ID | Description | GeneRatio | BgRatio | pvalue | p.adjust | qvalue | geneID | Count |
| --- | --- | --- | --- | --- | --- | --- | --- | --- | --- | --- |
| Organismal Systems | Immune system | hsa04612 | Antigen processing and presentation | 100% | 80/8844 | 6.21E-09 | 3.21E-08 | 8.71E-09 | HLA-C/HLA-DRB5/HLA-DQA1/HLA-DQB1 | 4 |
| Cellular Processes | Transport and catabolism | hsa04145 | Phagosome | 100% | 157/8844 | 9.56E-08 | 3.71E-07 | 1.01E-07 | HLA-C/HLA-DRB5/HLA-DQA1/HLA-DQB1 | 4 |
| Environmental Information Processing | Signaling molecules and interaction | hsa04514 | Cell adhesion molecules | 100% | 157/8844 | 9.56E-08 | 3.71E-07 | 1.01E-07 | HLA-C/HLA-DRB5/HLA-DQA1/HLA-DQB1 | 4 |
| Organismal Systems | Immune system | hsa04672 | Intestinal immune network for IgA production | 75% | 49/8844 | 6.37E-07 | 1.65E-06 | 4.47E-07 | HLA-DRB5/HLA-DQA1/HLA-DQB1 | 3 |
| Organismal Systems | Immune system | hsa04658 | Th1 and Th2 cell differentiation | 75% | 92/8844 | 4.33E-06 | 8.94E-06 | 2.43E-06 | HLA-DRB5/HLA-DQA1/HLA-DQB1 | 3 |
| Organismal Systems | Immune system | hsa04640 | Hematopoietic cell lineage | 75% | 99/8844 | 5.40E-06 | 9.59E-06 | 2.60E-06 | HLA-DRB5/HLA-DQA1/HLA-DQB1 | 3 |
| Organismal Systems | Immune system | hsa04659 | Th17 cell differentiation | 75% | 108/8844 | 7.02E-06 | 1.15E-05 | 3.11E-06 | HLA-DRB5/HLA-DQA1/HLA-DQB1 | 3 |

Supplementary table 6. Results of KEGG analysis.

| #node1 | node2 | node1_string_id | node2_string_id | neighborhood_on_chromosome | gene_fusion | phylogenetic_cooccurrence | homology | coexpression | experimentally_determined_interaction | database_annotated | automated_textmining | combined_score |
| --- | --- | --- | --- | --- | --- | --- | --- | --- | --- | --- | --- | --- |
| HLA-C | HLA-DQA1 | 9606.ENSP00000365402 | 9606.ENSP00000339398 | 0 | 0 | 0 | 0 | 0.081 | 0.126 | 0.54 | 0.87 | 0.945 |
| HLA-DQA1 | HLA-DRB5 | 9606.ENSP00000339398 | 9606.ENSP00000364114 | 0 | 0 | 0 | 0.65 | 0.322 | 0.471 | 0.65 | 0.331 | 0.904 |
| HLA-DQA1 | HLA-DQB1 | 9606.ENSP00000339398 | 9606.ENSP00000364080 | 0 | 0 | 0 | 0.637 | 0.812 | 0.989 | 0.54 | 0.947 | 0.999 |

Supplementary table 7. Results of PPI analysis.

| ID | Description | GeneRatio | BgRatio | pvalue | p.adjust | qvalue | geneID | Count |
| --- | --- | --- | --- | --- | --- | --- | --- | --- |
| PALLADIUM | PALLADIUM | 3月6日 | 12/19647 | 3.48E-09 | 1.22E-07 | 5.49E-08 | HLA-DRB5/HLA-DQA1/HLA-DQB1 | 3 |
| azathioprine | azathioprine | 2月6日 | 123/19647 | 0.000574 | 0.010039 | 0.004529 | HLA-DRB5/HLA-DQA1 | 2 |
| Insulin | Insulin | 2月6日 | 191/19647 | 0.001374 | 0.016036 | 0.007234 | HLA-C/HLA-DRB5 | 2 |
| N-Acetylmuramyl-L-alanyl-D-isoglutamine | N-Acetylmuramyl-L-alanyl-D-isoglutamine | 1月6日 | 16/19647 | 0.004877 | 0.036751 | 0.016579 | HLA-DRB5 | 1 |
| MERCURY | MERCURY | 2月6日 | 418/19647 | 0.0064 | 0.036751 | 0.016579 | HLA-DQA1/HLA-DQB1 | 2 |
| abacavir | abacavir | 1月6日 | 27/19647 | 0.008218 | 0.036751 | 0.016579 | HLA-C | 1 |
| NSC321521 | NSC321521 | 1月6日 | 28/19647 | 0.008522 | 0.036751 | 0.016579 | HLA-DQA1 | 1 |
| 2,4-Diisocyanato-1-methylbenzene | 2,4-Diisocyanato-1-methylbenzene | 1月6日 | 32/19647 | 0.009734 | 0.036751 | 0.016579 | HLA-DQB1 | 1 |
| 9-Anthroic acid | 9-Anthroic acid | 1月6日 | 37/19647 | 0.011248 | 0.036751 | 0.016579 | HLA-DRB5 | 1 |
| 6-Mercaptopurine | 6-Mercaptopurine | 1月6日 | 38/19647 | 0.01155 | 0.036751 | 0.016579 | HLA-DQA1 | 1 |
| Beryllium sulfate | Beryllium sulfate | 1月6日 | 38/19647 | 0.01155 | 0.036751 | 0.016579 | HLA-C | 1 |
| Inosinic acid | Inosinic acid | 1月6日 | 48/19647 | 0.014571 | 0.042478 | 0.019163 | HLA-DRB5 | 1 |
| Zebularine | Zebularine | 1月6日 | 52/19647 | 0.015778 | 0.042478 | 0.019163 | HLA-DQB1 | 1 |
| SODIUM SULFATE | SODIUM SULFATE | 1月6日 | 65/19647 | 0.019689 | 0.049223 | 0.022206 | HLA-DRB5 | 1 |

Supplementary table 8. Results of drug enrichment analysis.
